# Retraction: ‘ASPM predicts poor prognosis and regulates cell proliferation in bladder cancer’. Zhen‐Ya Gao, Fang Yu, Huan‐Xia Jia, Zhuo Ye, Shi‐Jie Yao, Kaohsiung J Med Sci. 2020; 36: 1021–1029 (https://doi.org/10.1002/kjm2.12284)

**DOI:** 10.1002/kjm2.12825

**Published:** 2024-03-28

## Abstract

The above article, published online on 06 August 2020, in Wiley Online Library (wileyonlinelibrary.com), has been retracted by agreement between the authors, the journal Editor‐in‐Chief, Wan‐Long Chuang, and John Wiley and Sons Australia, Ltd.

The retraction has been agreed due to a high degree of similarity and duplication of figures previously published in five identified articles. The authors are unable to determine how the images published were copies of figures from other articles.

Following the investigation by the Editors, the conclusions of this article are considered unreliable due to the high degree of duplication and the questionable origin of the data.

The above article, published online on 06 August 2020, in Wiley Online Library (wileyonlinelibrary.com), has been retracted by agreement between the authors, the journal Editor‐in‐Chief, Wan‐Long Chuang, and John Wiley and Sons Australia, Ltd.

The retraction has been agreed due to a high degree of similarity and duplication of figures previously published in five identified articles. The authors are unable to determine how the images published were copies of figures from other articles.

Following the investigation by the Editors, the conclusions of this article are considered unreliable due to the high degree of duplication and the questionable origin of the data.

